# A Health and Wellness Passport for Hispanic/Latino Informal Caregivers to Persons With ADRD

**DOI:** 10.1093/geroni/igaf122.720

**Published:** 2025-12-31

**Authors:** Shelly Aboagye

**Affiliations:** Marymount University, Arlington, Virginia, United States

## Abstract

The mental and functional health of Hispanic/Latino informal caregivers for older adults with cognitive impairments, such as Alzheimer’s disease and related dementias (ADRD), is a growing concern. Nearly 20% of American adults serve as informal or unpaid caregivers (Jeffers et al., 2021). However, the mild cognitive impairment that caregivers of these patients experience has not been thoroughly explored. According to the Centers for Disease Control and Prevention (CDC) Behavioral Risk Factor Surveillance System (BRFSS), a cross-sectional survey on subjective cognitive decline (SCD) among caregivers over the age of 45 in 22 states, 11% of the 1,434 caregivers surveyed in Virginia reported experiencing SCD between 2015 and 2019 (Jeffers et al., 2021). Furthermore, the cognitive function of Hispanic/Latino caregivers is adversely affected by stress and caregiver burden. To address this issue, we plan to develop a pilot study aimed at bridging the gap between caregiver needs and the efforts to address those needs. This study will evaluate the effectiveness and cultural sensitivity of a customized Health and Wellness Passport (Pasaporte de la Salud) specifically designed for Hispanic/Latino caregivers. The passport will include personalized health assessments, educational materials, and wellness resources. Evaluating the Health and Wellness Passport program will help determine its effectiveness and whether it reduces caregiver burden. This initiative will provide a deeper understanding of how to support vulnerable, at-risk Hispanic/Latino caregivers and promote their mental, physical, and functional well-being. Additionally, we will assess the practicality and usefulness of the bilingual (English and Spanish) Health and Wellness Passports.

